# Foramen Magnum Variant With Elongation of the Anterior Notch

**DOI:** 10.7759/cureus.8506

**Published:** 2020-06-08

**Authors:** Somya Bhatnagar, Joe Iwanaga, Tess Decater, Marios Loukas, R. Shane Tubbs

**Affiliations:** 1 Neurosurgery, Tulane Center for Clinical Neurosciences, Tulane University School of Medicine, New Orleans, USA; 2 Anatomical Sciences, St. George’s University, St. George's, GRD; 3 Anatomical Sciences, St. George's University, St. George, GRD; 4 Neurosurgery and Structural & Cellular Biology, Tulane University School of Medicine, New Orleans, USA; 5 Anatomical Sciences, St. George's University, St. George's, GRD; 6 Neurosurgery and Ochsner Neuroscience Institute, Ochsner Health System, New Orleans, USA

**Keywords:** foramen magnum, variant, anatomy, skull base, cadaver

## Abstract

Morphological variations of the foramen magnum (FM) have been demonstrated to have different shapes and sizes, according to sex, age, and ethnicity. In this report, an ancient Roman skull was found to have a unique anterior notching further specified as an anterior elongation of the FM. To our knowledge, this feature has not been previously reported. The FM is one of the most challenging neurosurgical regions due to both its deep location and proximity to vital structures. Therefore, physicians and surgeons must account for FM anatomical variations in order to properly diagnose craniocervical pathology, interpret radiological images, and optimize surgical outcomes. In this case report, we describe the possible embryology and clinical importance of an apparently rare FM variant.

## Introduction

The foramen magnum (FM) is the largest foramen of the skull, located in the posterior cranial fossa of the occipital bone. It marks the transition of the central nervous system between the spinal cord and brain. The spinal cord, vertebral arteries, anterior and posterior spinal arteries, meninges, and spinal roots of the accessory nerve pass through it. The craniocervical junction (CCJ) develops embryologically when the notochord induces neuroectodermal differentiation. The paraxial mesoderm contributes to the formation of bone and muscle in this region. The basin, median point of the anterior margin of the FM, is derived from the proatlas. The elongation of the clivus and anterior FM results from lateral sutural growth. The bone around the FM results from descent of the occiput and growth of the petro-occipital and sphenopetrosal junctions. The FM size and area is determined by the endochondral parts of the basiocciput, exocciput, and supraocciput and the interoccipital synchondroses between them [1,2]. The FM is surrounded by the pars lateralis, pars squama, and pars basilaris components of the occipital bone. 

A unique FM with anterior notching and elongation was found in an ancient Roman skull. This case demonstrates the unique morphologies of the FM. Its location is important as it is near the fourth ventricle, lower cranial nerves (IX-XII) and upper cervical nerves (C1-C2), and medulla oblongata [3]. Surgeons should understand the FM and surrounding region in order to prevent injury of structures passing through it and avoid complications during procedures.

## Case presentation

**Figure 1:**
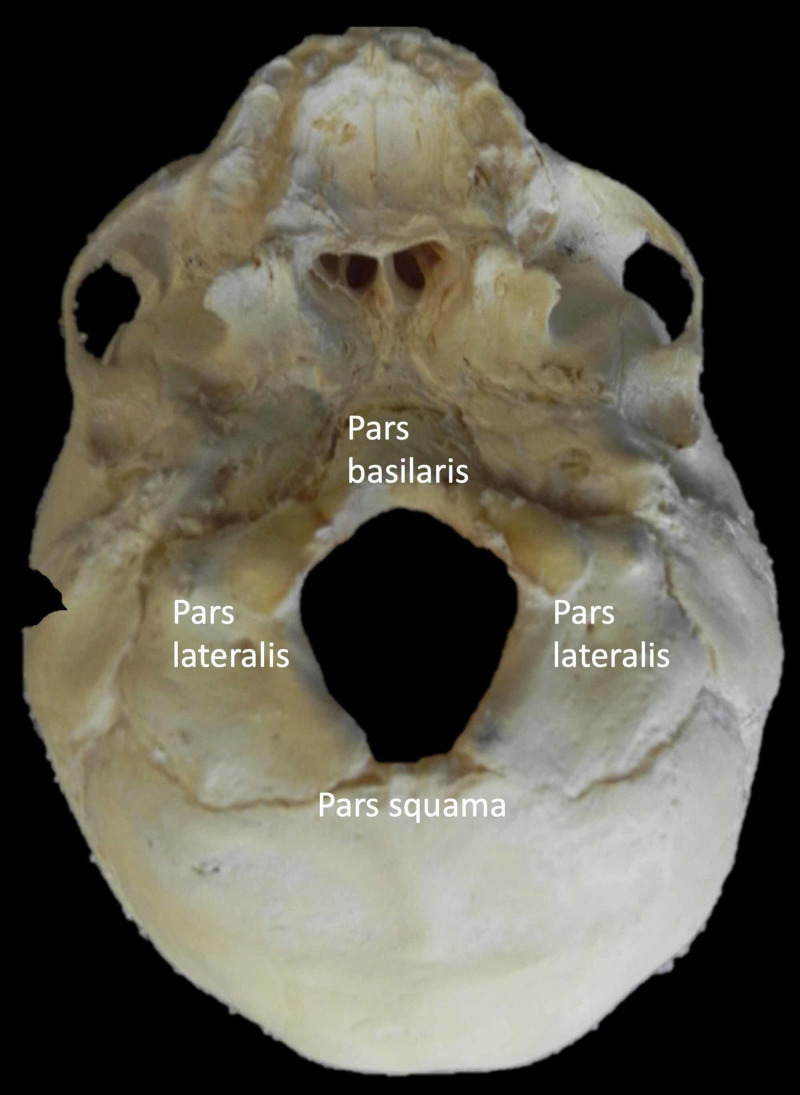
Skull base of an infant noting the parts of the occipital bone Note the developing parts of the occipital bone surrounding the foramen magnum.

**Figure 2:**
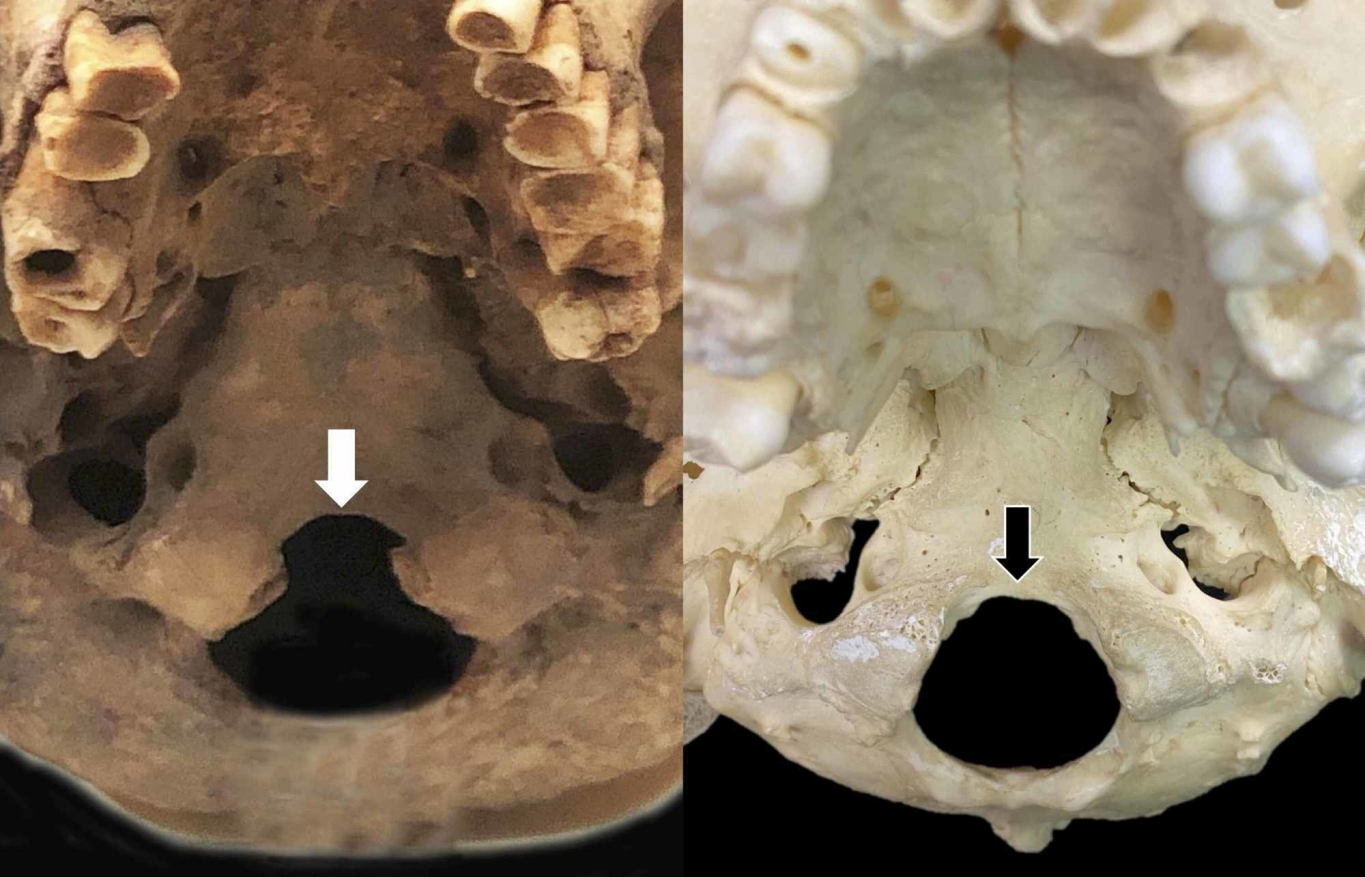
A unique foramen magnum (FM) with anterior notching (white arrow) in an ancient Roman skull (left) For comparison, the normal FM without such an anterior notch (black arrow) is shown (right). Note the extension of the FM beyond the borders of the occipital condyles, which is out of the ordinary.

A unique FM with anterior notching was found in ancient roman skull (Figures 1, 2)

 

The skull was that of an adult male. From an inferior view, the anterior notch of the FM was significantly elongated. The width of the FM was considered normal at about 27 mm, but the length was considerably longer than normal at approximately 40 cm (normal 30 cm) [1]. Additionally, this variant anterior part of the FM extended anterior to the occipital condyles almost to the level of the hypoglossal canal. No other anatomical variants were noted at the skull base in this specimen. The left and right occipital condyles were within normal limits in regard to position and size. The dimensions of the clivus were found to be within normal limits. The adjacent supraocciput and basiocciput were found to be within normal limits. The adjacent jugular foramina did not exhibit any anatomical variations.

## Discussion

We presented a very unusual case where the FM extended beyond the position of the occipital condyles with an elongated anterior segment. Normally, the FM does not extend beyond the borders of the occipital condyles.

The shape of the FM has many named variants, such as rhomboid, circle, heart, pear, and hexagon, although these names are inconsistent between studies. The FM may also be asymmetrical, or there may be a different degree of protrusion of the occipital condyles into it [4]. Aragão et al. described the most predominant morphological types of FM as pear-shaped, rounded, and tetragonal [5]. Samara et al. concluded that an irregularly shaped FM was most predominant in analysis of a Jordanian population [6]. However, the main type varies significantly in the literature due to inconsistent shape labels.

Aside from interstudy discrepancies in FM shape labeling, variations in FM morphology are often due to sexual dimorphism (size) and different ethnic groups (shape). Zdilla et al. described males as having a longer sagittal length and larger FM area than females, but no significant difference in shape. The shape of the East Asian FM was closest to the average FM shape, followed by Europeans and Bengalis. The African, Bengali, and Malayan populations tended to have a more elongated FM shape [2].

Another determinant of variation in the shape of the FM is age. Lang described five different shapes of the FM: two semicircles, elongated circle, egg-shaped, rhomboid, and rounded. They compared them based on prevalence in two age groups: adults and children [7]. It was reported that the most common shape in the adult age group was two semicircles (41.2%), and the most common shape in the children age group was rhomboid (31.6%). These results illustrate the importance in considering age when considering the FM.

The variation in FM shape occurs postnatally. During the fetal period, the characteristic shape of the FM is a long oval shape, likely due to the 5.4% faster growth speed in the sagittal direction compared to the transverse direction between the seventh month in utero and birth [2]. During postnatal growth, the FM shape becomes more variable. Between birth and sixth months of age, growth of the FM in the transverse direction is 7.6% greater than growth in the longitudinal direction [2]. Also occurring at birth, the ventral part of the FM widens due to an increased growth rate of the anterior interoccipital synchondroses, causing the ventral FM to invaginate more deeply into the basiocciput [8]. The elongation of the anterior notch of the FM seen in the present case may result from such growth. 

Variations in the shape and size of the FM are also relevant in forensic and anthropological studies as this structure’s dimensions are used to determine the sex, age, ethnicity, stature, and other important identifying information of an individual. 

Conditions such as achondroplasia (small FM) and Chiari malformations (specifically Chiari type II malformations) (often large FM) demonstrate the variation in FM size [8]. In achondroplasia, the reduced size and shape restrict CSF egress through the FM, leading to potential hydrocephalus. In contrast, in Chiari malformations, the cerebellar vermis protrudes through the enlarged FM. However, this protrusion is thought to precede the formation of the FM. Significant elongation of the ventral FM would efface the posterior pharynx [4]. 

The variation in the shape of the FM is important to consider for neurosurgeons. Avci et al. described that with an ovoid FM, it is more challenging for a surgeon to access its anterior portion [9]. The degree of protrusion of the occipital condyles into the FM is also a factor. In the ovoid type of FM, more extensive bony removal of the occipital condyles may be required, whereas condylectomies with shorter occipital condyles may cause occipitocervical instability. Thus, the anterior elongation of the FM could affect surgical access to the anterior FM such as for resection of meningiomas or access to other structures of the CCJ such as in odontoidectomy. Additionally, such a variant might give surgeons more working space to resect tumors of this region without removing the condyles [10]. 

## Conclusions

The surgeon and clinician should keep anatomical variations of the FM in mind when making preoperative decisions or diagnosing patients. Anatomical knowledge of variations of FM might enable surgeons to read radiography such as computed tomography more accurately at prediagnostic stage.
